# Back to basics: likelihood ratios for olive and grass pollen specific IgE in seasonal allergic rhinitis

**DOI:** 10.3389/falgy.2023.1241650

**Published:** 2023-10-04

**Authors:** Bárbara Manzanares, Rafael González, Pilar Serrano, Ana Navas, Corona Alonso, Lourdes Fernandez, Aurora Jurado, Carmen Moreno-Aguilar

**Affiliations:** ^1^ Maimonides Biomedical Research Institute of Córdoba (IMIBIC), Reina Sofia University Hospital/ University of Córdoba, Córdoba, Spain; ^2^ Department of Immunology and Allergy, Reina Sofia University Hospital, Córdoba, Spain; ^3^ Postdocs CSYF, Code RH-0060-2020, European Social Fund, Sevilla, Spain; ^4^ National Network ARADyAL, Health Institute Carlos III, Madrid, Spain

**Keywords:** seasonal allergic rhinitis/asthma, allergen immunotherapy, likelihood ratios, Olea, Poaceae, cut-off values

## Abstract

**Introduction:**

Specific IgE (sIgE) is merely a sensitization marker that cannot be used for allergy diagnosis if there are no associated clinical symptoms. As of 2023, there is still no evidence regarding the quantity of sIgE necessary to confirm or exclude clinical disease. Therefore, this study aimed to calculate cut-offs for sIgE, allowing us to effectively diagnose olive or grass pollen allergy and select allergenic immunotherapy (AIT) candidate patients in a region under high olive and grass allergenic pressure.

**Methods:**

An observational retrospective study consisting of the review of electronic medical records from 1,172 patients diagnosed with seasonal rhino-conjunctivitis and suspected allergy to olive or grass pollen. Symptoms correlated with sIgE to Poaceae and Oleaceae whole extracts and sIgE to genuine allergenic components were evaluated. Optimal cut-off values were calculated using receiver operating characteristic curves. Relevant clinical symptoms and AIT indications were taken into consideration when determining the clinical allergy diagnosis.

**Results:**

sIgE to Lolium showed the best area under the curve (AUC) for both diagnosis (0.957) and an indication of AIT (0.872). The optimal cut-off values for grass diagnosis and AIT indication were 1.79 kUA/L and 8.83 kUA/L, respectively. A value of 5.62 kUA/L was associated with a positive likelihood ratio (LR) of 10.08 set for grass allergy. Olea sIgE showed the best AUC for the diagnosis (0.950). The optimal cut-off for diagnosis was 2.41 kUA/L. A value of 6.49 kUA/L was associated with a positive LR of 9.98 to confirm olive pollen allergy. In regard to immunotherapy, Ole e 1 sIgE showed the best AUC (0.860). The optimal cut-off was 14.05 kUA/L. Ole e 1 sIgE value of 4.8 kUA/L was associated with a 0.09 negative LR to exclude olive AIT indication.

**Conclusions:**

The sIgE cut-offs found in this population under high olive and grass allergenic pressure reduce the gap between sensitization and clinical allergy, providing a new tool for the diagnosis of seasonal allergic rhinitis/asthma and helping to discriminate patients who will benefit from AIT.

## 1. Introduction

Allergy-mediated airway diseases are a major health concern due to their high prevalence, increasing incidence, and clinical manifestations affecting multiple organs. According to the World Health Organization (WHO), 500 million people worldwide suffer from rhinitis and 262 million have asthma. These two diseases significantly impair the quality of life of the inflicted individuals and their relatives (1).

Nowadays, allergy specialists have no tools to distinguish between sensitization and clinical allergy by just using *in vitro* testing (2, 3).

Disease confirmation, identification of the triggers (allergens), and severity assessment are key to determining the need and appropriateness of therapies aimed to modify the course of the disease, such as allergen-specific immunotherapy.

For an accurate diagnosis, quantification of sIgE is a decisive factor. However, the current criteria for determining positive/negative or clinically relevant/non-relevant sensitization/non-sensitization are influenced by a lack of evidence-based cut-offs (4).

The “clinically relevant sensitization” concept, generally considered as sIgE detectable loads in the context of consistent symptoms, is a must to discriminate which patients would benefit from allergen immunotherapy. Besides the traditional optimal cut-off based on sensitivity/specificity balance, a new diagnostics strategy based on the ratios of whole extract-specific IgE/total IgE and component-specific IgE/whole-extract specific IgE has recently been proposed to define the relevance of sensitization in the patient symptoms (5).

Differentiating mono from polysensitized patients is also relevant for their management. Among allergic pollens, olive tree pollen (*Olea europaea*) is one of the most important respiratory allergy triggers in the Mediterranean area and in regions of America, South Africa, Japan, and Australia (6). In Spain, it is considered the second cause of pollinosis, and the first in certain southern regions. To date, 14 allergenic proteins have been identified in olive pollen, from Ole e 1 to Ole e 12, Ole e 14, and recently Ole e 15 (7) (according to the nomenclature of the International Union of Immunological Societies (IUIS, International Union of Immunological Societies) (8, 9). In addition to olive, grass pollen is another relevant pollen in Mediterranean countries, and in fact, both share overlapping pollination periods, thus increasing the diagnostic complexity in areas where they coexist. Due to this overlap, it is difficult to determine whether symptoms are due to genuine sensitization or cross-reaction. Therefore, a component-resolved diagnosis (CRD) is needed to establish the genuine sensitizer(s).

Double sensitization to Poaceae and Oleaceae makes it more challenging when evaluating the indication of AIT. In this case, the specific allergenic component culprits of the two-pollen species must be identified to discriminate between genuine bisensitization and cross-reactivity, thus allowing the specialists to pinpoint the specific trigger(s) (i.e., grass and/or olive pollen) (3, 10–13).

In conclusion, our aim was to establish effective and efficient cut-off points for sIgE to genuine allergenic components and to the whole allergens (Poaceae and Oleaceae). By establishing these cut-off points, we could identify genuine sensitizers and ultimately predict the clinical reactivity to these pollens while increasing the probability of AIT success in highly exposed patients.

## 2. Materials and methods

### 2.1 Design and study population

Retrospective study carried out in Hospital Reina Sofía, Córdoba, (Spain, south area). The study population included all consecutive patients referred for allergy evaluation to our hospital from January 2015 to April 2018. Inclusion criteria were established as suspicion of olive and/or grass pollen allergy according to general practitioner anamnesis.

**Figure 1:**
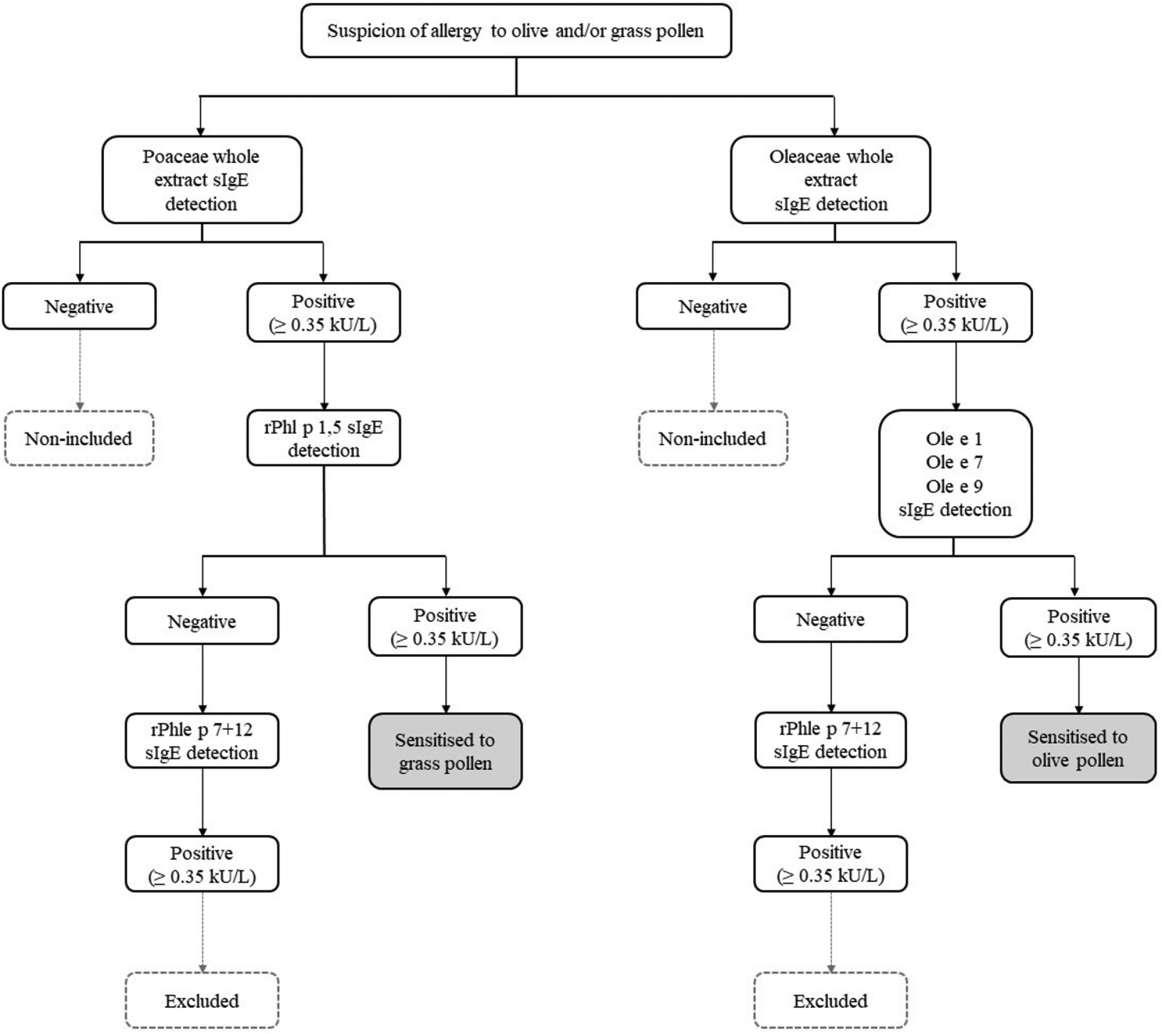
Diagnostic algorithm used to rule in grass and/or olive pollen sensitization. When sIgE to grass (rPhl p 1,5) and olive (ole e 1, ole e 7, and ole e 9) pollens molecular components were negative, sIgE to Phleum polcalcin (rPhl p 7) and Phleum profilin (rPhl p 12) (cross-reactive markers) were determined. If positive results were obtained, patients were excluded from the study.

At the first visit, all included patients were subjected to skin prick testing with grass/olive pollen extracts. Patients with negative results for both pollens were then excluded. For patients displaying a positive result or negative result (but with high clinical suspicion), specific IgE (sIgE) tests were requested as detailed in the diagnostic algorithms (Figure 1).

According to our proposed algorithm, corresponding to our routine testing, the first step was sIgE testing for Poaceae (*Lolium perenne* whole extract) and Oleaceae (*Olea europea* whole extract) pollen antibody loads. Then, if the result was positive (>0.35 kU_A_/L), the sIgE levels for olive (Ole e 1, Ole e 7 and Ole e 9) and grass (Phl p 1 and Phl p 5 (rPhl p 1,5) allergen genuine sensitization components were tested. For those sera testing negative, sIgE levels to highly cross-reactive allergens (polcalcins (rPhl p 7) and profilins (rPhl p 12)) were determined (Figure 1). Finally, if sIgE was detected for one or both panallergens, the patients were excluded (14–17).

All clinical information (anamnesis, skin test results, sIgE loads, and final diagnosis) was collected from the patient's electronic medical records. Based on this information, the allergy specialists evaluated the appropriateness of allergen-specific immunotherapy according to standard guidelines for AIT prescription.

A total of 1,205 cases were initially included. However, 33 of them were excluded, and therefore a total of 1,172 cases were finally considered for the statistical analysis.

### 2.2 Data collection

Clinical data and laboratory marker values were obtained from electronic medical records corresponding to the clinical episode at the inclusion visit.

#### 2.2.1. Clinical variables

The collection form included demographic, epidemiological, and clinical data such as age at diagnosis, gender, symptoms (rhino-conjunctivitis, asthma), skin tests, laboratory data, clinical diagnosis, and immunotherapy details. Patients with rhino-conjunctivitis were classified following ARIA guidelines (18) as mild or moderate-severe; patients suffering from asthma were classified according to the Spanish guidelines on asthma management “GEMA” (19) as mild, moderate, or severe. The results of skin tests (positive/negative), sIgE determinations (kU_A_/L), final diagnosis (allergic/non-allergic), and consideration of immunotherapy (yes/no) were also recorded.

Outcome variables were the final diagnosis and AIT indication.

#### 2.2.2. Skin prick tests (SPTs)

Skin prick tests (SPTs) were performed following the routine diagnostic procedure, using available commercial extracts: *Olea europaea*, *Lolium perenne,* and profilin (ALK Abelló, Madrid, Spain). Polcalcin extract was supplied by ALK Abelló as no commercial extract. A positive result was established for a mean diameter wheal of ≥3 mm (20). The results were expressed as positive/negative for each of the tested extracts.

#### 2.2.3. Total serum IgE, and sIgE levels

Serum total IgE (tIgE), sIgE to the whole extracts, and species-specific components from olive and grass pollen were tested. Total serum IgE levels were measured by sandwich immunoassay on an Advia Centaur analyser (Siemens Healthcare, USA); sIgE load to Lolium, Olea, rPhl p 1,5, rPhl p 7 + 12, Ole e 1, Ole e 7, and Ole e 9 were measured by fluoroenzymeimmunoassay with ImmunoCAP (Thermo Fisher Scientific, Uppsala, Sweden) on a Phadia 250 system, according to the manufacturer's specifications.

#### 2.2.4. Allergological diagnosis

The allergological diagnosis was established through the integration of clinical data, SPT results, and sIgE values, according to allergy specialist criteria for clinical routine. Patients with clinically relevant symptoms of rhinitis/rhinoconjunctivitis/bronchial asthma during the olive or grass pollen pollination period (April to June) and positive sIgE (Prick-test or ImmunoCAP) were considered allergic (21).

#### 2.2.5. Aetiological treatment

Immunotherapy indication was considered for allergic patients with moderate-severe or poorly controlled rhinitis. In the case of bronchial asthma, it was indicated when the disease was well controlled with low or medium levels of treatment [therapeutic steps 2–4 from GEMA (19)]. The composition was decided based on clinical data and test results, under the judgment of an allergist according to the standard clinical practice conditions. Subsequently, patients received this treatment according to their indications, contraindications (22), or personal circumstances (difficult accessibility, unwillingness to comply, high cost).

### 2.3. Statistical analysis

The Kolmogorov-Smirnov test was used to assess the normal data distribution. Demographic and clinical characteristics of patients were expressed as mean and standard deviation (SD); If not adjusting for a normal distribution, the median was used to represent non-parametric data for continuous variables, and frequency distributions represented categorical variables.

The Mann–Witney test or Kruskal–Wallis test was used to analyse the relationship between continuous and categorical variables. Spearman's correlation coefficient was used to evaluate the correlation between quantitative variables.

The diagnostic validity of the sIgE loads to each allergen was determined by considering as outcome variables the clinical diagnosis and the indication for AIT. For this purpose, sensitivity, specificity, positive predictive value (PPV), negative predictive value (NPV), the receiver operating curve (ROC), and the area under the curve (AUC) were calculated with the following comparisons:

1. Grass allergy or both vs. non-allergic or Olive allergy.
2. Olive allergy or both vs. non-allergic or Grass allergy

Finally, the maximum value of the Youden Index (YI) was used as a criterion for selecting the optimal cut-off value for each variable (23). Moreover, the positive and negative likelihood ratios were calculated to maximise the specificity (to rule in allergy or AIT indication) and the sensitivity (to rule out allergy or AIT indication) (24).

## 3. Results

### 3.1. Characteristics of the study population

A total of 1,172 individuals with suspected allergy to olive or grasses were included. Descriptive baseline characteristics of the total population and stratified according to final allergy diagnosis are shown in Tables 1–5. Included subjects ranged from 16 to 37 years, with a median of 24 years. Women represented 57%. Study variables (age, serum tIgE, and sIgE levels) showed a non-Gaussian distribution. Most patients presented mild asthma, and the percentage of patients with mild or moderate to severe rhinoconjunctivitis was roughly the same. The median IgE load was 193.5 IU/ml for tIgE, 5.5 kU_A_/L for Olea-sIgE, and 3.5 kU_A_/L for Lolium sIgE. 70.5% of the included subjects were positive for olive extract and 58.4% positive for grass extract using SPT. According to the final diagnosis made by the Allergy specialist, patients were classified into four groups: 17.8% (209) were diagnosed with grass allergy, 24% (281) with olive allergy and 29.2% (341) were allergic to both pollens; moreover, allergy to olive/grass pollen was ruled out in 29.1% (341) of the individuals. AIT was indicated in 29.6% of the patients. The final percentage of patients who received AIT was 22.3%.

**Table 1:** Baseline characteristics of allergic and non-allergic individuals.

Clinical and demographic characteristics	All patients *n* = 1,172 (%)			
Gender				
Male	503 (42.9)			
Female	669 (57.1)			
Skin tests				
Grass positive	638 (58.4)			
Olive positive	771 (70.5)			
Profilin positive	163 (15.1)			
Polcalcin positive	118 (12.1)			
Allergy symptoms				
Rhinoconjunctivitis				
No	81 (6.9)			
Mild	560 (47.8)			
Moderate/severe	531 (45.3)			
Asthma				
No	257 (21.9)			
Mild	564 (48.1)			
Moderate-severe	351 (29.9)			
Immunotherapy				
No	906 (77.7)			
Grass and olive immunotherapy	58 (5.0)			
Only olive immunotherapy	118 (10.1)			
Only grass immunotherapy	85 (7.3)			
	Mean	Median	SD*	IQR^†^
Age	26.4	24	13.6	16–37
Total IgE (kU/L)	443.6	193.5	673.7	74.2–495.3
Lolium sIgE^‡^(kU_A_/L)	21.4	3.5	52.8	0.2–17.4
rPhl p 1,5 sIgE (kU_A_/L)	20.5	4.7	43.2	0.5–19.4
Olea sIgE (kU_A_/L)	49.8	5.5	109.7	0.5–42.7
Ole e 1 sIgE (kU_A_/L)	34.4	4.6	81.4	0.6–26.1
Ole e 7 sIgE (kU_A_/L)	26.8	0.5	67.0	0.1–14.9
Ole e 9 sIgE (kU_A_/L)	14.7	0.0	43.7	0.0–3.4

SD*, standard deviation; IQR^†^, interquartile range; sIgE^‡^, specific Immunoglobulin E.

**Table 2:** Baseline characteristics of grass-allergic patients.

Clinical and demographic characteristics	All patients *n* = 209 (%)			
Gender				
Male	86 (41.1)			
Female	123 (58.9)			
Skin tests				
Grass positive	187 (93.0)			
Olive positive	111 (55.2)			
Profilin positive	49 (24.9)			
Polcalcin positive	27 (14.2)			
Allergy symptoms				
Rhinoconjunctivitis				
No	6 (2.9)			
Mild	106 (50.7)			
Moderate-severe	97 (46.4)			
Asthma				
No	46 (22.0)			
Mild	118 (56.5)			
Moderate-severe	45 (21.5)			
Immunotherapy				
No	124 (59.3)			
Grass immunotherapy	85 (40.7)			
	Mean	Median	SD*	IQR^†^
Age	28.7	28	12.0	19–37.5
Total IgE (kU/L)	252.3	142.5	295.1	59.0–301.5
Lolium sIgE^‡^(kU_A_/L)	39.4	19.0	56.9	7.8–47.8
rPhl p 1,5 sIgE (kU_A_/L)	34.3	15.1	48.8	5.4–42.3

SD*, standard deviation; IQR^†^, interquartile range; sIgE^‡^, specific Immunoglobulin E.

**Table 3:** Baseline characteristics of olive-allergic patients.

Clinical and demographic characteristics	All patients *n* = 281 (%)			
Gender				
Male	139 (49.5)			
Female	142 (50.5)			
Skin tests				
Grass positive	84 (32.6)			
Olive positive	252 (97.7)			
Profilin positive	19 (7.4)			
Polcalcin positive	18 (8.1)			
Allergy symptoms				
Rhinoconjunctivitis				
No	2 (0.7)			
Mild	123 (43.8)			
Moderate/severe	156 (55.5)			
Asthma				
No	26 (9.3)			
Mild	135 (48.0)			
Moderate-severe	120 (42.7)			
Immunotherapy				
No	173 (61.6)			
Olive immunotherapy	108 (38.4)			
	Mean	Median	SD*	IQR^†^
Age	22.8	21.0	13.2	13.0–32.5
Total IgE (kU/L)	646.1	326.0	834.0	135.0–757.0
Olea sIgE^‡^ (kU_A_/L)	88.7	38.9	122.3	12.9–99.9
Ole e 1 sIgE (kU_A_/L)	55.5	16.6	100.5	4.3–55.9
Ole e 7 sIgE (kU_A_/L)	44.7	7.6	82.4	0.4–57.4
Ole e 9 sIgE (kU_A_/L)	20.3	0.0	54.1	0.0–11.0

SD*, standard deviation; IQR^†^, interquartile range; sIgE^‡^, specific Immunoglobulin E.

**Table 4:** Baseline characteristics of grass and olive allergy.

Clinical and demographic characteristics	All patients *n* = 341 (%)			
Gender				
Male	159 (46.6)			
Female	182 (53.4)			
Skin tests				
Grass positive	301 (94.7)			
Olive positive	309 (96.9)			
Profilin positive	91 (29.1)			
Polcalcin positive	65 (23.8)			
Allergy symptoms				
Rhinoconjunctivitis				
No	9 (2.6)			
Mild	153 (44.9)			
Moderate/severe	179 (52.5)			
Asthma				
No	38 (11.1)			
Mild	174 (51.0)			
Moderate/severe	129 (37.8)			
Immunotherapy				
No	271 (79.9)			
Grass and olive immunotherapy	58 (17.1)			
Only olive immunotherapy	10 (3.0)			
Only grass immunotherapy	0 (0.0)			
	Mean	Median	SD*	IQR^†^
Age	24.3	23.0	12.3	15–32.3
Total IgE (kU/L)	604.6	323.3	726.6	145.0–787.0
Lolium sIgE^‡^(kU_A_/L)	41.0	14.7	74.7	6.1–41.6
rPhl p 1,5 sIgE (kU_A_/L)	28.5	10.7	51.5	3.8–29.4
Olea sIgE (kU_A_/L)	84.1	30.4	145.2	8.2–84.3
Ole e 1 sIgE (kU_A_/L)	43.4	11.4	89.1	2.9–43.1
Ole e 7 sIgE (kU_A_/L)	31.1	0.9	72.1	0.2–22.4
Ole e 9 sIgE (kU_A_/L)	20.8	0.2	48.1	0.0–15.9

SD*, standard deviation; IQR^†^, interquartile range; sIgE^‡^, specific Immunoglobulin E.

**Table 5:** Baseline characteristics of non-allergic individuals.

Clinical and demographic characteristics	All patients *n* = 341 (%)			
Gender				
Male	119 (34.9)			
Female	222 (65.1)			
Skin tests				
Grass positive	66 (20.9)			
Olive positive	99 (31.3)			
Profilin positive	4 (1.3)			
Polcalcin positive	8 (2.7)			
Allergy symptoms				
Rhinoconjunctivitis				
No	64 (18.8)			
Mild	178 (52.2)			
Moderate/severe	99 (29.0)			
Asthma				
No	147 (43.1)			
Mild	137 (40.2)			
Moderate-severe	57 (16.7)			
Immunotherapy				
No	338 (100)			
Grass and/or olive immunotherapy	0 (0)			
	Mean	Median	SD*	IQR^†^
Age	30.1	29.0	15.0	18–41
Total IgE (kU/L)	239.8	82	526.2	33.8–232.5
Lolium sIgE^‡^(kU_A_/L)	1.3	0.1	6.9	0.0–0.5
rPhl p 1,5 sIgE (kU_A_/L)	3.0	0.4	12.4	0.0–1.2
Olea sIgE (kU_A_/L)	0.8	0.1	3.5	0.0–0.6
Ole e 1 sIgE (kU_A_/L)	1.2	0.3	4.6	0.0–0.8
Ole e 7 sIgE (kU_A_/L)	0.7	0.0	4.8	0.0–0.1
Ole e 9 sIgE (kU_A_/L)	0.1	0.0	0.3	0.0–0.0

SD*, standard deviation; IQR^†^, interquartile range; sIgE^‡^, specific Immunoglobulin E.

### 3.2. Specific IgE

**Figure 2:**
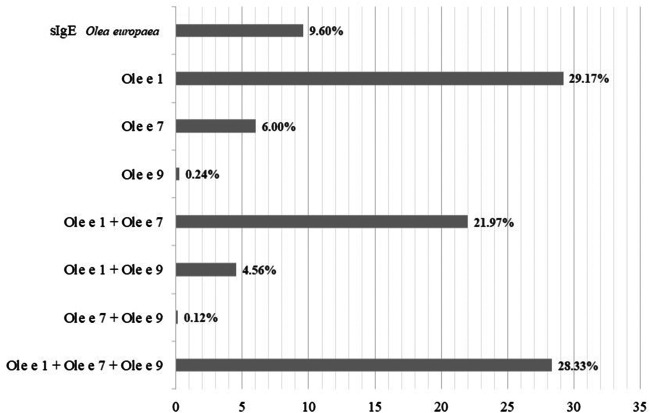
Sensitization profiles were found in the study group according to the serum sIgE levels to whole extract of *olea europaea* pollen and its molecular components, considering 0.35 kU_A_/L as the positive cut-off value.

sIgE values to the whole olive and grass extracts and their respective molecular components are shown in Tables 1–5. Among patients sensitized to Olea according to the standard cut-off (≥0.35 kU_A_/L), 84% were positive to Ole e 1, 56.4% were positive to Ole e 7, and, 33.4% were positive to Ole e 9. Figure 2 sIgE patient profiles. Almost all patients with Ole e 9 sIgE loads above the standard cut-off were also positive for Ole e 1 (98.6%). However, only 37.2% of Ole e 7 positive were also Ole e 1 positive. sIgE loads to Olea pollen showed a Spearman's correlation coefficient with Ole e 1, Ole e 7, and Ole e 9 of 0.849 (*p* < 0.001), 0.818 (*p* < 0.001) and 0.636 (*p* < 0.001) respectively. For Ole e 1 and Ole e 9, the correlation was 0.628 (*p* < 0.001). Ole e 1 and Ole e 7 showed a correlation of 0.585 (*p* < 0.001). Finally, the correlation between Ole e 7 and Ole e 9 was 0.496 (*p* < 0.001). Moreover, the correlation coefficient for Ole e 1 and Ole e 9 in patients Ole e 9 positive was 0.706. Regarding grasses, Lolium sIgE and rPhl p 1,5-sIgE showed an overall Spearmańs correlation coefficient of 0.904 (*p* < 0.001).

### 3.2. Immunotherapy

85 from 209 (40.7%), 118 from 281 (42%), and 58 from 341 (17%), grass pollen allergic patients, olive or both respectively, received AIT (Table 1). None of the non-allergic patients received immunotherapy.

### 3.3. Diagnostic validity

Univariate ROC analysis considering grass allergy final diagnosis (Lolium monosensitization or in combination with olive pollen allergy), Lolium sIgE showed the best area under the curve (AUC 0.957; 95% CI 0.945–0.968). Thus, the optimal Lolium-sIgE cut-off was 1.79 kU_A_/L with 98.47% sensitivity and 82.77% specificity. Moreover, a value of 5.62 kU_A_/L showed a positive likelihood ratio (+LR) of 10.08, corresponding to 92.27% specificity 92.27%. Considering the grass allergy indications as the result variable, once again, Lolium showed the best AUC (0.872: 95% CI 0.849–0.896). Nevertheless, considering rPhl p 1,5-sIgE as final variables showed also relevant results. The optimal Lolium cut-off for grass AIT indication was 8.83 kU_A_/L with 93.98% sensitivity and 71.94% specificity (Table 6).

**Table 6:** Diagnostic accuracy of sIgE.

For grass allergy		AUC*	AUC 95% IC^†^	+LR^‡^	-LR^§^	YI^∥^	Sn^¶^ (%)	Sp** (%)
**Lolium extract**		0.957	0.945–0.968					
Standard cut-off	0.35			2.4	0.00	0.58	100	58.42
Best cut-off and Cut-off for -LR	1.79			5.72	0.02	0.81	98.47	82.77
Cut-off for +LR	5.62			10.08	0.24	0.7	77.86	92.28
**rPhl p 1,5**		0.933	0.916–0.951					
Standard cut-off	0.35			2.5	0.04	0.58	97.25	61.07
Best cut-off and cut-off for -LR	1.60			5.3	0.09	0.75	92.49	82.55
Cut-off for +LR	5.10			10.08	0.31	0.64	71.06	92.95
**For olive allergy**		** **	** **			** **		** **
**Olea extract**		0.95	0.936–0.963					
Standard cut-off	0.35			2.00	0.00	0.5	100	50.1
Best cut-off and Cut-off for -LR	2.41			5.85	0.06	0.79	95.35	83.7
Cut-off for +LR	6.50			9.99	0.19	0.74	82.39	91.75
**Ole e 1**		0.882	0.858–0.906					
Standard cut-off	0.35			1.86	0.12	0.426	93.83	49.66
Best cut-off	1.74			4.73	0.18	0.67	85.23	81.97
Cut-off for +LR+	10.45			10.11	0.48	0.5	55.03	94.56
Cut-off for -LR	None				na^††^			
**Ole e 7**		0.864	0.837–0.891					
Standard cut-off	0.35			3.89	0.36	0.52	70.29	81.95
Best cut-off	0.08			2.87	0.14	0.59	90.26	68.59
Cut-off for +LR	5.65			10.08	0.59	0.39	43.67	95.67
Cut-off for -LR	0.05			2.24	0.08	0.53	95.29	57.4
**Ole e 9**		0.724	0.691–0.757					
Standard cut-off	0.35			7.01	0.6	0.37	43.65	93.67
Best cut-off	0.18			5.64	0.57	0.39	47.56	91.58
Cut-off for +LR	0.47			9.86	0.59	0.39	43.32	95.6
Cut-off for -LR	None				na			
For grass immunotherapy								
**Lolium extract**		0.87	0.849–0.896					
Standard cut-off	0.35			1.49	0.00	0.33	100	33.11
Best cut-off and Cut-off for -LR	8.83			3.35	0.08	0.66	93.98	71.94
Cut-off for +LR	187.00			10.42	0.90	0.10	10.53	98.99
**rPhl p 1,5**		0.85	0.816–0.874					
Standard cut-off	0.35			1.38	0.03	0.27	99.3	28.12
Best cut-off	8.36			2.88	0.14	0.59	90.14	68.72
Cut-off for +LR	None				na			
Cut-off for -LR	4.61			2.30	0.10	0.53	94.37	58.97
For olive immunotherapy								
**Olea extract**		0.856	0.831–0.880					
Standard cut-off	0.35			1.37	0.00	0.27	100	26.75
Best cut-off	22.4			3.45	0.21	0.6	83.83	75.72
Cut-off for +LR	None				na			
Cut-off for -LR	5.42			2.25	0.09	0.53	94.61	57.93
**Ole e 1**		0.86	0.834–0.886					
Standard cut-off	0.35			1.32	0.02	0.24	99.43	24.86
Best cut-off	14.05			3.55	0.22	0.6	83.33	76.5
Cut-off for +LR	None				na			
Cut-off for -LR	4.80			2.42	0.09	0.55	94.25	61.07
**Ole e 7**		0.679	0.642–0.716					
Standard cut-off	0.35			1.73	0.33	0.35	82.86	52.1
Best cut-off	0.19			1.69	0.2	0.37	90.86	46.36
Cut-off for +LR	None			na				
Cut-off for -LR	0.1			1.53	0.11	0.33	96	37.11
**Ole e 9**		0.741	0.698–0.784					
Standard cut-off	0.35			2.51	0.5	0.37	62.07	75.32
Best cut-off	2.77			3.11	0.52	0.39	57.47	81.52
Cut-off for +LR	None				na			
Cut-off for -LR	None				na			
**Ole e 1/Ole e 7**		0.664	0.618–0.711					
Best cut-off	58.19			2.94	0.68	0.28	41.95	85.74
Cut-off for +LR	224.33			10.61	0.9	0.1	10.92	98.97
Cut-off for -LR	None				na			

AUC*, Area under the curve; AUC 96% IC^†^, 95% Confidence Interval of the area under the curve; +LR^‡^, positive likelihood ratio; -LR^§^, negative likelihood ratio; YI^∥^, Youden Index; Sn^¶^, Sensitivity; Sp**, Specificity; na^††^, non-applicable.

Univariate ROC analysis considering olive pollen allergy final diagnosis (Olea monosensitization or in combination with grass pollen allergy), Olea sIgE showed the best area under the curve (AUC 0.950; 95% CI 0.936–0.963). The optimal Olea sIgE cut-off was 2.41 kU_A_/L, with 95.34% sensitivity and 83.70% specificity (Table 6). Moreover, an Olea sIgE value of 6.49 kU_A_/L showed a +LR of 9.98, providing strong certainty for olive allergy (specificity 91.75%). Considering the AIT indication for olive allergy as the result variable, Ole e 1-sIgE showed the best AUC (0.86; 95% CI 0.83–0.88). The optimal Ole e 1 cut-off for olive AIT was 14.05 kU_A_/L, with 83.33% sensitivity and 76.5% specificity. Ole e 1 load of 4.8 kU_A_/L showed a -LR of 0.09 to exclude olive AIT (sensitivity 94.25%). The diagnostic accuracy threshold for each allergen is shown in Table 6.

### 3.4. Diagnostic validity of the proposed cut-off with actual data

We compared -in our population- the performance of the standard cut-off (0.35 kU_A_/L) with the best cut-off values obtained with our analysis, where 2.4 kU_A_/L for Olea-sIgE and 1.79 kU_A_/L for Lolium-sIgE for clinical diagnostic purposes. Likewise, we compared the performance of the standard cut-off (0.35 kU_A_/L) with the best cut-off for Ole e 1-sIgE (14.05 0.35 kU_A_/L) and Lolium-sIgE (8.83 0.35 kU_A_/L) obtained from our analysis for indication of AIT. The statistical parameters, sensitivity, specificity, and positive and negative predictive values are shown in Table 7.

**Table 7:** Diagnostic and AIT prescription accuracy for sIgE* with newly established cut-offs.

(A) Diagnostic accuracy according to cut-off values					
	Cut-off	Sn^†^	Sp^‡^	PPV^§^	NPV^∥^
Olea extract	0.35	100	50.1	70.82	100
	2.41	95.3	83.7	87.63	93.69
Lolium extract	0.35	100	58.01	71.19	100
	1.79	98.66	82.37	85.31	98.34
(B) Immunotherapy prescription accuracy according to cut-off values					
	Cut-off	Sn	Sp	PPV	NPV
Ole e 1	0.35	99.42	24.86	23.92	99.45
	14.05	83.33	76.5	45.74	95.07
Lolium extract	0.35	100	32.88	18.19	100
	8.83	93.98	71.94	33.33	98.76

sIgE*, specific Immunoglobulin E; Sn^†^, Sensitivity; Sp^‡^, Specificity; PPV^§^ Positive predictive value; NPV^∥^, Negative predictive value.

### 3.5. Symptoms and sIgE

**Figure 3:**
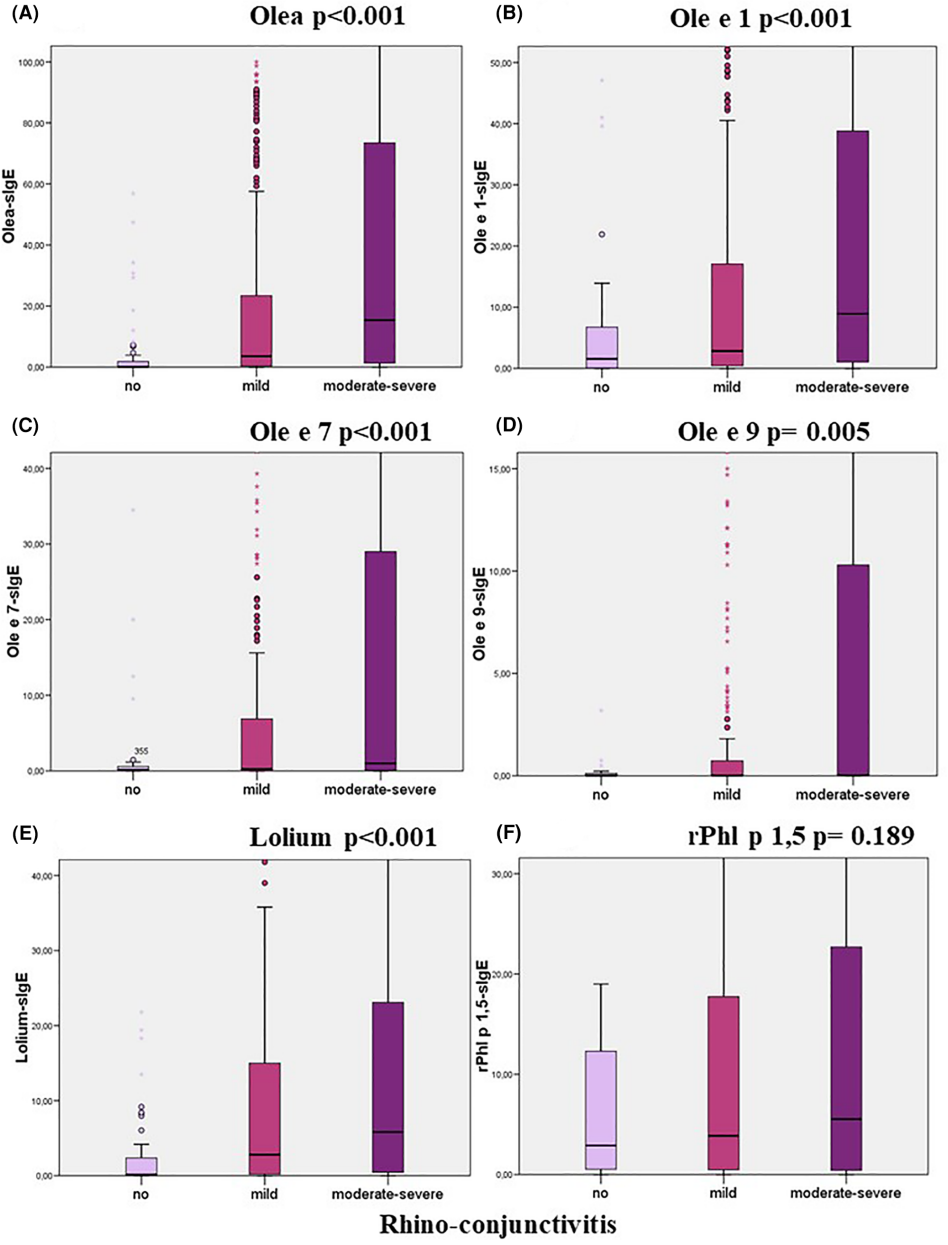
Relationship between specific IgE levels and the degree of rhino-conjunctivitis categorised according to the ARIA guidelines (Kruskal–Wallis test).

**Figure 4:**
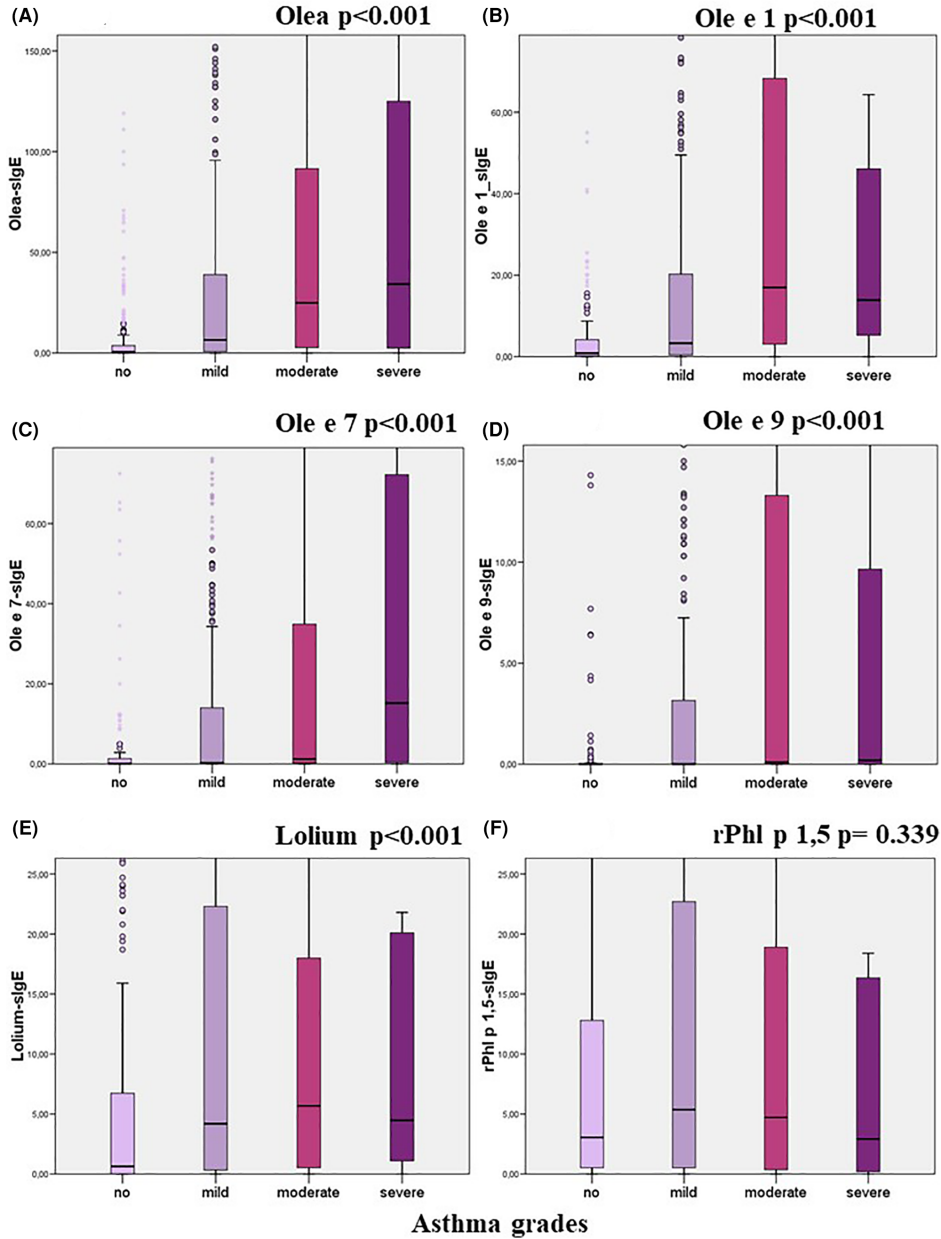
Relationship between specific IgE levels and the degree of asthma categorised according to the GEMA guidelines (Kruskal–Wallis test).

**Figure 5:**
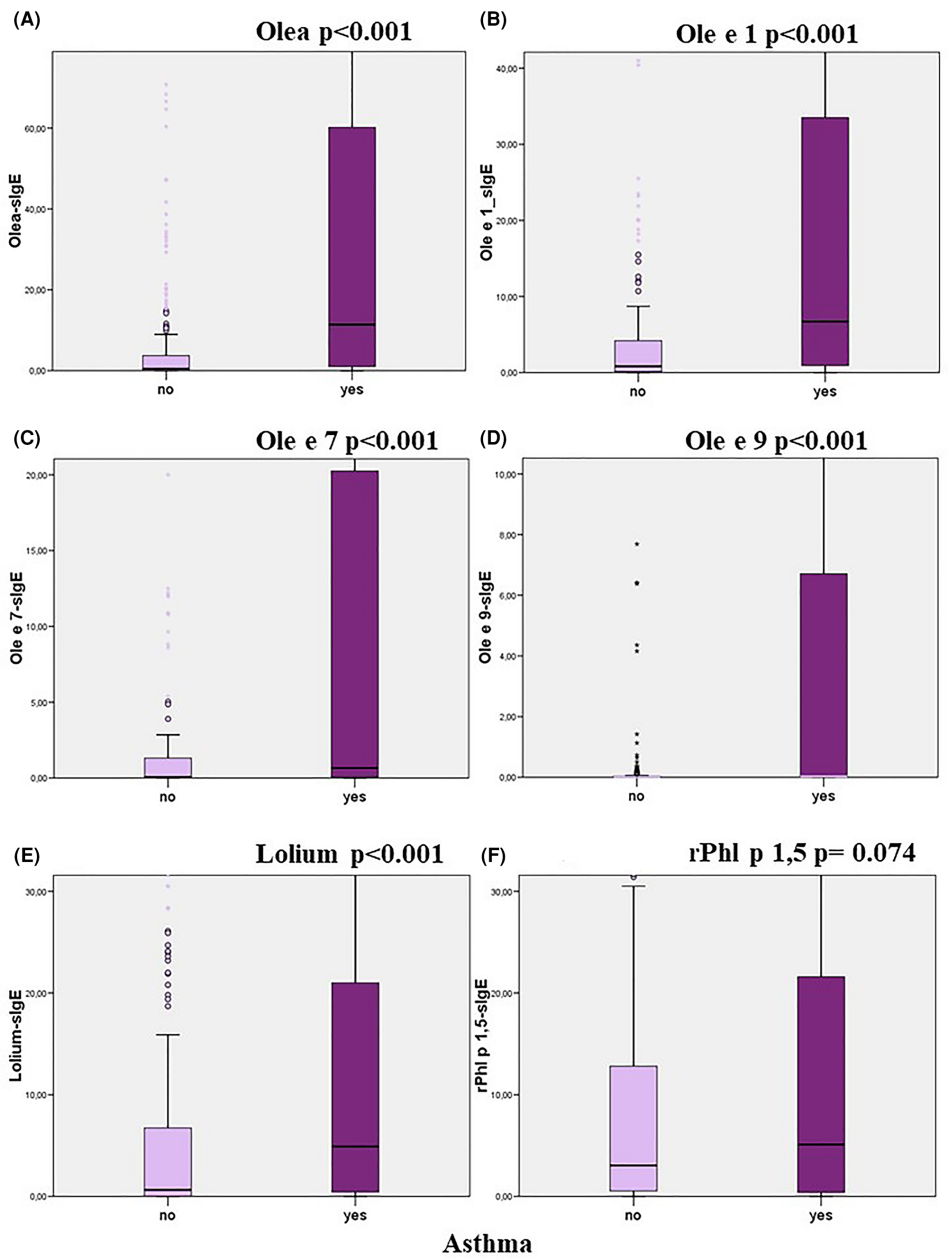
Relationship between specific IgE levels and the presence of asthma (Mann–Witney test).

The relationship between the levels of sIgE to each studied allergen and the presence of symptoms (Mann-Whitney) and its severity (Kruskal–Wallis) were analysed. Higher loads of sIgE to Olea, Ole e 1, Ole e 7, Ole e 9, and Lolium were associated with the presence or severity of rhino-conjunctivitis and/or asthma (Table 8; Figures 3–5). However, this association was not found to be significant for the levels of rPhl 1,5-sIgE.

**Table 8:** Correlation between allergic symptoms and sIgE* levels.

Symptoms	Olea extract	Ole e 1	Ole e 7	Ole e 9	Lolium extract	rPhl p 1,5
Rhinoconjunctivitis (no, mild, moderate-severe)	<0.001	<0.001	<0.001	0.005	<0.001	0.189
Asthma (no, mild, moderate, severe)	<0.001	<0.001	<0.001	<0.001	<0.001	0.331
Asthma (yes, no)	<0.001	<0.001	<0.001	<0.001	<0.001	0.074

sIgE*, specific Immunoglobulin E.

## 4. Discussion

Diagnosing allergic asthma and/or rhinoconjunctivitis is a complex task. Although serological tests are helpful, they all require interpretation from an allergy specialist. Of 1,172 patients for whom allergy to two olive and/or grass pollen was suspected, 831 were confirmed as clinical allergic patients to one or both. Not all the 341 patients for whom allergy was ruled out tested negative for sIgE if considering 0.35 kU_A_/L as the cut-off. Thus, revealing the lack of real validated cut-offs to assess the sensitization clinical relevance. The sIgE cut-off has been generally set to 0.35 kU_A_/L, without considering other factors that could influence the immunogenicity of the allergen and the characteristics of the disease presentation. For the same reason, values below this cut-off have been shown to be relevant (25). This arbitrary cut-off has been under debate in different publications that, using different approaches, have tried to find cut-offs for sIgE aligned with clinical reality, both for diagnosis (26–28) and treatment (indication of AIT or provocation tests, both of them with side effects) (4, 29–31). Few studies have been published specifically looking for these cut-offs in the diagnosis of seasonal rhinitis and or asthma to date (32, 33). This work aimed to obtain cut-offs for sIgE in our population under high olive and grass allergenic pressure to help us in the decision to indicate AIT.

The simultaneous pollination of olive trees and grasses increases the diagnostic complexity in our area and therefore, more improved diagnostic tools are required, such as the establishment of evidence-based cut-offs and discrimination between mono vs. polysensitization. The proposed algorithm allows us to discriminate while minimizing the number of sIgE tests (Figure 1).

Cut-off evaluation analysis has revealed that the AUC of Lolium-sIgE shows a very good performance for this whole extract, and is very similar to the one corresponding to rPhl p 1,5-sIgE. The same trend can be seen when looking for the best cut-off. Likewise, the correlation between Lolium-sIgE and rPhl p 1,5-sIgE was excellent, as expected (14–17). Given the above, we understand that the allergy diagnosis can be made efficiently with either of the two Poaceae determinations without expanding the algorithm. Due to cross-reactivity of profilins and polcacins (34), we better recommend using the determination of rPhl p 1,5-IgE for the diagnosis, considering the cut-off above 1.65 kU_A_/L in our population. When the best grass was obtained, the cut-off was applied to our patient samples, and good sensitivity (92.49%) and much better specificity (82.55%) were obtained compared to those obtained with the 0.35 kU_A_/L cut-off (Table 7). Schäfer et al. evaluated the diagnostic value of SPT and sIgE in 75 children with hay fever and concluded that although at 0.35 kU_A_/L NPV was similar for both methods (close to 100%), the PPV was very low for the *in vitro* assay. Increasing ImmunoCAP cut-off to 1.5 kU_A_/L paired PPV from both methods (32). Van Hoeyveld et al., aimed to study sIgE cut-offs in pollen allergy, including grasses, using +LR as the main diagnostic tool. They concluded that for grass and birch pollen, the likelihood ratios for allergy increased with sIgE load. The likelihood ratio was <0.03 for specific IgE <0.1 kU_A_/L, between 1.4 and 4.2 for sIgE between 0.35 kU_A_/L and 3.5 kU_A_/L, and very high (∞) for specific IgE >3.5 kU_A_/L (33).

There are currently no consensus criteria for indication of AIT to grass pollen according to sIgE levels. In our daily practice, AIT is only prescribed in grass patients with Lolium-sIgE levels above 8.83 kU_A_/L. This value is considerably higher than the diagnostic cut-off, considering that the prescription of grass AIT depends on several other factors. Thus, only patients with a considerable quality of life impairment due to allergic symptoms are selected for AIT. In our study, the severity of rhino-conjunctivitis and asthma correlated with Lolium sIgE levels, but paradoxically, not with those of rPhl p 1,5. It can be explained by the fact that severity has been shown to increase together with the number of recognized components, which may be represented in the Lolium extract (35, 36).

Unlike grasses, Olive pollen contains a greater diversity of genuine and relevant allergenic components in our region, and different sensitization profiles (Figure 2) need to be considered (37). The use of defined diagnostic algorithms is appropriate depending on the outcome that we want to achieve. If the clinical goal is merely confirmatory (confirmation of true clinical allergy), the determination of Olea sIgE is appropriate, using the cut-off of 6.50 kU_A_/L to diagnose the allergy with confidence. However, if there is an additional objective such as immunotherapy prescription or patient phenotyping for any other purpose, it is essential to modify the algorithm. In this specific case, we recommend starting from the 2.41 kU_A_/L cut-off.

The use of component-resolved extended diagnosis for olive pollen allergy depends, again, on the therapeutic goal (38). In cases where a most comprehensive diagnosis is required, it makes sense to implement an automated algorithm when established criteria are met. In our experience, it does not seem appropriate to test for components if Olea sIgE is less than 2.41 kU_A_/L. When AIT is considered, the only determination of Olea sIgE in our population may be insufficient, even at levels above 6.50 kU_A_/L, since Ole e 1, Ole e 7, and Ole e 9 display different sensitization profiles (Figure 2), as shown previously (12). Clinical symptoms and AIT response will differ depending on which one of them is the dominant sensitizer. For example, AIT is not recommended in patients with olive pollen allergy mainly driven by Ole e 7. Given the variability of the allergenic content of the different immunotherapy extract industrial batches and the AIT-related adverse event incidence, this must be considered (39). Diagnosis based on the ratio between sIgE to the molecular component and the sIgE to the whole extract may be appropriate to determine the main allergy driver component (5).

In our population, Ole e 1 sIgE above 10.45 kU_A_/L was confirmatory for clinical allergy to olive pollen, but it was not a necessary condition. In other words, low or even undetectable levels of Ole e 1 sIgE did not rule out the diagnosis of olive pollen allergy, which could also be confirmed by Ole e 7 sIgE using the same diagnostic approach: values above 5.65 kU_A_/L are also confirmatory. Both Ole e 1 and Ole e 7 have similar correlation coefficients with Olea whole extract. Ole e 9 showed a lower value because its presence is almost always associated with Ole e 1 sIgE.

When the best Olea sIgE cut-off was applied to our population, a good sensitivity (95.3%) and an increased specificity (83.7%) (Table 7) were obtained in comparison to those achieved with the arbitrary 0.35 kU_A_/L cut-off.

The fact that we were unable to settle a cut-off value for Olea AIT indication reinforces the concept that this is a multiple factor-dependent decision that must be made on a case-by-case basis. A real-life example is represented by Ole e 9, which does not seem to influence the indication for olive AIT in our population. Nevertheless, the evidence of variability in Ole e 9 content from one immunotherapy batch to another requires an accurate characterization of Ole e 9 sensitized patients and only commercial immunotherapy extracts containing considerable amounts of the protein are valid for them (39).

Moreover, the Ole e 7 cut-offs for AIT prescription that we have calculated are biased by a particular prescribing habit in our group, which in clinical practice reduces the prescription of olive pollen AIT to patients exhibiting much higher levels of sIgE Ole e 7 than sIgE Ole e 1. The main reason is that a higher incidence of AIT systemic reactions in olive-allergic patients with exclusive or predominant sensitisation to Ole e 7 has been described (40). In fact, current EAACI recommendations do not recommend AIT treatment for patients with Ole e 7 sensitisation (41).

As also described by Moreno et al. (37), it should be noted that the final indication for AIT is conditioned by the sensitization complexity, meaning that it does not only depend on the different components involved but also the relative amount of sIgE to each of them. This issue was particularly relevant in the 58 patients who received double AIT (olive pollen extract plus grass pollen extract).

The main limitation of this study is the retrospective nature of data from clinical practice without a specific previous design. For this reason, there are notable data gaps, such as tIgE in some included patients. The analysis of the so-called ratios and the determination of cut-off values for these ratios could also have been of interest to complete our studies (5). Another limitation is that the sIgE values used in the ROC curves for diagnosis and immunotherapy probably conditioned the expert's decision, both in the diagnostic conclusion and the indication for immunotherapy. In addition, the response to specific immunotherapy was not assessed in this study, and therefore, there are no data to test the goodness of ROC curves to predict this response. Finally, implementing the quality-of-life scales to assess the severity of asthma/rhinitis could have introduced relevant information for our analysis.

Allergic profiles are local, so when talking about the generalization of the results (use of our established cut-off in other clinical settings), we must consider that this study was carried out in a single center in a specific geographical area; therefore, the safety of the data obtained when applied in other regions, still has to be determined. Nevertheless, the value of the differences in the variables between groups has been obtained with an explicitly stated confidence interval of 95%.

The main strength of the present study is that it thoroughly examines the use of likelihood ratios for the diagnosis and the immunotherapy indication in olive and grass pollen allergy. The reporting of sIgE sensitization *in vitro* test results using a Bayesian approach has recently gained interest. In contrast to the dichotomy of a single, often arbitrary cut-off point, the use of LRs helps to determine to what extent a test result changes the probability of a particular diagnosis or treatment indication (32, 42–45). Hence, the present study could help allergists in clinical decision-making. In any case, this work would reflect the need to establish different cut-off points for the standards used, which would interest other centers.

## 5. Conclusions

This study establishes different cut-offs (supported by robust data from a big patient cohort) that shorten the gap between sensitization and clinical relevance in the diagnosis of seasonal allergic rhinitis to olive or grass pollen, or both. It also provides cut-offs that, together with the clinical history and the sensitization profile, could help in clinical decision-making, such as the AIT prescription. Additionally, it suggests that simplified diagnostic algorithms would save analytical determinations while improving patient diagnosis and healthcare system efficiency.

Further prospective studies are needed in which the predictive value of sIgE for diagnosis could be compared with the expert's diagnosis, blinded to this factor and based on clinical and skin prick test results. Likewise, it could be helpful to include the evaluation of the response to immunotherapy and not exclusively the indication for immunotherapy.

## Data Availability

The raw data supporting the conclusions of this article will be made available by the authors, without undue reservation.
